# 妊娠合并抗凝血酶Ⅲ缺乏症1例报告并文献复习

**DOI:** 10.3760/cma.j.cn121090-20241126-00483

**Published:** 2024-12

**Authors:** 桃英 李, 赛赛 王, 明丽 周, 梓然 张, 宝菊 朱

**Affiliations:** 郑州大学第二附属医院产科，郑州 450000 Department of Obstetrics, the Second Affiliated Hospital of Zhengzhou University, Zhengzhou 450000, China

**Keywords:** 抗凝血酶Ⅲ缺乏, 华法林, 静脉血栓形成, 妊娠结局, Antithrombin Ⅲ deficiency, Warfarin, Venous thrombosis, Pregnancy outcome

## Abstract

**目的:**

报道1例妊娠合并抗凝血酶（antithrombin，AT）Ⅲ缺乏症孕妇的诊断和治疗，结合文献梳理诊治思路。

**方法:**

回顾2023年10月至2024年6月郑州大学第二附属医院收治的1例AT-Ⅲ缺乏症孕妇的临床诊治过程，并结合相关文献，总结妊娠合并AT-Ⅲ缺乏症对妊娠结局的影响，并为抗凝方案的制定提供参考。

**结果:**

患者有3次不良孕产史及双下肢深静脉血栓史，本次妊娠期AT活性波动于34％～50％，孕期共输新鲜冰冻血浆3次，孕前开始使用低分子肝素至分娩前24 h，孕中期加用华法林、羟氯喹、环孢素及甲泼尼龙，孕35周终止妊娠，胎儿出生体重2 330 g，早产儿转入儿科治疗14 d出院，随访至今未发现异常。

**结论:**

对妊娠合并AT-Ⅲ缺乏症给予精准治疗可有效改善妊娠结局。

## Abstract (en)

**Objective:**

To report the diagnosis and treatment of a pregnant woman with antithrombin（AT）Ⅲ deficiency in pregnancy, and combine the diagnosis and treatment ideas with the literature.

**Methods:**

The clinical diagnosis and treatment process of one pregnant woman with AT-Ⅲ deficiency admitted to the Second Affiliated Hospital of Zhengzhou University from October 2023 to June 2024 were retrospectively analyzed. Combined with relevant literature, the effect of pregnancy with AT-Ⅲ deficiency on pregnancy outcome was summarized, and reference was provided for the formulation of anticoagulant regimen.

**Results:**

The patient had 3 adverse pregnancy histories and a deep venous thrombosis of the lower limbs. The AT activity fluctuated between 34％ and 50％ during this pregnancy. The patient received 3 transfusions of fresh frozen plasma during pregnancy and was administered low molecular weight heparin from pre-pregnancy until 24 hours before delivery. Warfarin, hydroxychloroquine, cyclosporine and methylprednisolone were added in the second trimester. The pregnancy was terminated at 35 weeks of gestation. The infant was born with a weight of 2,330 g and was transferred to the pediatric department for treatment. After 14 days, the infant was discharged. Follow-up to date has not revealed any abnormalities.

**Conclusion:**

Precise treatment of pregnancy with AT-Ⅲ deficiency can effectively improve pregnancy outcomes.

人体出凝血平衡涉及六大系统，包括凝血、抗凝、纤溶、血管内皮、血小板、血流动力学系统，各方相互调控、相互代偿，使出凝血处于动态平衡。抗凝系统以抗凝血酶系统和蛋白C系统（包括蛋白C、蛋白S）为主。抗凝血酶系统中抗凝血酶（antithrombin，AT）是人体最重要的抗凝因子之一，约占抗凝活性的70％[1]。AT缺乏症导致抗凝能力下降，加之妊娠期高凝状态，增加了妊娠期母体静脉血栓或母胎界面血栓的发生率，导致流产、早产、胎儿生长受限、胎盘早剥、子痫前期甚至死胎等不良妊娠结局发生。现将郑州大学第二附属医院收治的1例AT-Ⅲ缺乏症孕妇诊疗经过及妊娠结局报道如下，以提高临床医师对该病的认识及诊治能力。

## 病例资料

一、病例

患者，女，29岁，以“发现AT-Ⅲ缺乏6个月余，停经8周6天”为主诉于2023年10月23日就诊我院。2023年4月5日备孕时检测：AT活性为41％，同型半胱氨酸（HCY）13.85 µmol/L，亚甲基四氢叶酸还原酶（MTHFR）C677T CT型，蛋白C、蛋白S活性正常，抗磷脂抗体谱（含非典型磷脂抗体）正常，自身免疫抗体谱正常。予依诺肝素（0.6 ml q12h）、阿司匹林（50 mg qd）及活性叶酸治疗。本次妊娠自然受孕，平素月经规律，末次月经：2023年8月22日，早孕反应不明显，孕早期无腹痛或阴道出血等。孕8周2天（2023年10月19日）复查AT活性为34％、蛋白S 29％、蛋白C 84％，为提高AT活性收住院。既往1次剖宫产手术史、1次深静脉血栓史。孕4产1（G4P1），3次不良孕产史：G1：2016年孕34周无明显诱因出现“胎盘早剥”，外院急诊剖宫产并产后出血，娩一女活婴，出生体重1.7 kg（1.2百分位数），现体健；G2：2019年孕26周因“胎儿脐血流反向、胎动减少”外院住院，后“死胎”引产；G3：2020年孕26周因“胎动减少、羊水过少”外院住院，AT活性为21％，蛋白C、蛋白S活性正常，狼疮抗凝物筛选试验/确认试验比值（LAS-R/LAC-R）1.20/0.99，标准化LAC比值（NLR）1.21，后因“重度胎儿生长受限、羊水过少”引产娩一男死婴，产后未抗凝治疗，产后第10天双下肢深静脉血栓，插管溶栓术后口服华法林（3～9 mg qd），术后1个月因国际标准化比值（INR）仍未达标准，改口服利伐沙班（10 mg qd），药物治疗1年后自行停药至本次备孕。月经史：平素月经规律，13岁初潮，经期6天，月经周期30天，量正常、有血块。家族史：患者外婆有脑梗塞病史，父母、1弟1妹均体健，无遗传病家族史。

入院后查肝肾功能、血常规、凝血功能正常，超声提示孕囊及胎芽与孕周符合，宫腔积液（5.8 mm），双侧子宫动脉血流舒张期血流频谱消失，体重75 kg，身体质量指数26.5 kg/m^2^。于AT活性近30％的孕9周、12周4天、26周2天分别给予输注新鲜冰冻血浆（FFP）600 ml、400 ml、400 ml。首次血浆输注后AT活性上升12％，后续两次分别上升6％、7％。在依诺肝素持续抗凝基础上于孕19周6天加服华法林（3 mg qd），根据血凝指标（表1）逐渐调整剂量至6mg qd，但INR仍未达到《易栓症诊断与防治中国指南（2021年版）》[2]推荐的2～3范围内的治疗目标。

**表1:** 妊娠合并抗凝血酶Ⅲ缺乏症患者的凝血指标

孕周	9W	12W	15W1D	17W	20W1D^a^	21W	22W2D^b^	23W5D	28W1D	30W^c^	32W6D	34W6D^d^	术后13D	术后44D^e^	参考范围
PT（s）	13.1	13.5	13.3	13.6	16.5	23.3	15.5	14.4	17.2	24.4	21.0	13.4	12.9	14.0	11~14
APTT（s）	43.7	38.8	43.8	37.2	55.3	46.5	36.3	35.3	39.6	41.5	39.8	37.9	44.9	37.5	30~46
TT（s）	21.2	17.0	19.1	16.6	22.2	16.8	16.6	18.0	16.6	16.3	16.0	16.9	26.6	17.2	14~21
FIB（g/L）	2.60	2.64	3.24	2.93	3.10	3.42	3.48	3.29	3.36	3.30	3.79	3.81	2.73	2.41	2~4
D-Dimer（µg/ml）	0.20	0.57	0.31	0.48	–	0.49	0.29	–	–	0.46	0.51	0.92	–	–	0~0.5
INR	0.99	1.03	1.01	1.04	1.33	2.10	1.24	1.13	1.40	2.23	1.82	1.00	0.95	1.07	0.82~1.12
PLT（×10^9^/L）	130	211	–	158	196	–	165	191	–	141	140	105	199	188	125~350
R（min）	–	5.5	8.8	–	–	9.2	–	–	–	12.2	9.3	–	7.2		5~10
K（min）	–	1.8	2.8	–	–	1.9	–	–	–	3.2	2.2	–	1.9		1~3
Angle	–	65.20	52.2	–	–	62.7	–	–	–	49.9	71	–	63.1		53~72
MA（mm）	–	58.4	51.9	–	–	59.9	–	–	–	58.9	71.5	–	56.4		50~70

**注** W：周；D：天；PT：凝血酶原时间；APTT：活化部分凝血活酶时间；TT：凝血酶时间；FIB：纤维蛋白原；D-Dimer：D-二聚体；INR：国际标准化比率；PLT：血小板；R：凝血因子作用时间；K；血凝块形成时间；Angle：血凝块形成速率；MA：最大血凝块强度；9～17W为2023年度，20W后为2024年度；^a^开始启用华法林3 mg/d；^b^加入免疫抑制剂；^c^华法林从6 mg/d减量；^d^分娩、分娩后启用依诺肝素；^e^停用肝素一周；–：未检测

患者本次妊娠孕12～20周期间多次检测狼疮抗凝物均呈阳性（表2）；孕22周4天超声提示早发型重度胎儿生长受限（2.5百分位数），给予口服羟氯喹0.2 mg bid、甲泼尼龙4 mg qd、环孢素50 mg bid改善免疫等。通过以上治疗后胎儿生长发育趋于正常，孕30周因凝血酶原时间（PT）延长、血栓弹力图提示血液低凝，下调华法林用量至孕33周2天停药，同时行下肢血管超声提示：管腔内血流连续通畅。孕34周停服阿司匹林，依诺肝素用至分娩前24 h。孕35周剖宫产娩一女活婴，出生体重2 330 g（35.5百分位数）。新生儿因早产转新生儿重症监护病房，监测PT 33.9 s、INR 3.47、纤维蛋白原（FIB）<0.60 g/L，活化部分凝血活酶时间（APTT）95.3 s、凝血酶时间（TT）44.5 s，予FFP 60 ml输注后凝血功能恢复正常，给予无创辅助通气、抗感染、静脉营养及光疗等，产后14 d痊愈出院，随访至今未发现异常，产后继续使用依诺肝素抗凝至产后近6周。

**表2:** 妊娠合并抗凝血酶Ⅲ缺乏症患者的易栓症五项

孕周	AT-Ⅲ（％）	PC（％）	PS（％）	NLR	LAS-R	LAC-R	检测日期
8W2D	34	–	29	–	–	–	2023.10.19
10W1D	38	86	42	1.37	1.53	1.12	2023.11.13
13W3D	44	114	48	1.40	1.58	1.13	2023.11.24
16W6D	41	103	33	1.17	1.3	1.11	2023.12.18
18W3D	40	82	35	1.24	1.38	1.11	2023.12.29
19W6D	41	102	40	1.21	1.38	1.14	2024.01.08
22W2D	47	80	22	1.12	1.34	1.20	2024.01.25
23W5D	46	108	22	1.17	1.33	1.14	2024.02.04
26W5D	43	89	18	1.10	1.28	1.16	2024.02.25
28W1D	50	62	19	1.41	1.74	1.23	2024.03.06
31W6D	42	20	9	1.43	2.02	1.41	2024.04.01
34W1D	38	84	24	1.16	1.15	0.99	2024.04.17
34W6D	32	87	29	–	–	–	2024.04.22
参考范围	80～130	70～130	55～140	<1.2	<1.2	<1.2	

**注** W：周；D：天；AT-Ⅲ：抗凝血酶Ⅲ；PC：蛋白C；PS：蛋白S；NLR：标准化狼疮抗凝物比值；LAS-R：狼疮抗凝物筛选试验比值；LAC-R：狼疮抗凝物确认试验比值；–：未检测

二、基因测序及分析

1. 血液样本采集及基因测序：取得患者及家属同意后，两次采集该患者的外周静脉血4 ml，委托广州知力医学检验实验室进行高通量基因测序。

2. 生物信息学分析及基因致病性的判断：第1次检测包含58个血栓遗传基因，如PROC、PROS1、SERPINC1、ADAMTS13等，结果与遗传性AT缺乏不相符，同时提示血管性血友病因子（VWF）基因c.3550G>A:p.Asp1184Asn。进一步完善VWF活性和抗原水平检查，采用免疫比浊法，VWF活性：90.7％（参考范围49.5％～187.0％），血管性血友病因子抗原：81.1％（参考范围50.0％～160.0％）。凝固法监测凝血因子全套，提示Ⅷ因子活性明显降低（48.9％，参考范围70.0％～150.0％）、Ⅻ因子活性明显降低（45.3％，参考范围70.0％～150.0％）。

再次检测涵盖241个基因（表3），范围涉及血栓风险、出血倾向（凝血因子缺陷、血管性血友病等）、血小板功能异常。提示：VWF基因如前突变；ANKRD26基因3986c.A>C:p.Lys1329Thr；SERPINC1:c.1154-53G>A（rs2759328）和SERPINC1:c.41+141G>A（rs2227589）两个内含子杂合突变。患者的弟弟进行AT活性检测，结果正常（93％），基本排除遗传性AT缺乏症。

**表3:** 妊娠合并抗凝血酶Ⅲ缺乏症患者涵盖241个基因测序的结果

染色体	基因	基因突变位点	基因型	ExAC-EAS	ACMG证据级别
12	VWF	NM-000552:exon 27:c.35 50G>A:P.Asp1184Asn	杂合突变	/	临床意义不明 突变
10		NM-014915:exon28:398 6C.A>C:p.Lys1329Thr	杂合突变	0.0001	临床意义不明 突变
根据基因突变相关分级标准，良性突变不予报告			
1	SERPINCI	NM-000488.4:C.1154-53 G>A	内含子 杂合突变	/	良性突变
1	SERPINCI	NM-000488.4:c.41+141 G>A	内含子 杂合突变	/	良性突变

**注** /：未检测

## 讨论及文献复习

一、文献复习

以“妊娠合并抗凝血酶缺乏/缺陷”“Pregnancy complicated with antithrombin/deficiency”为关键词，检索获得相关中文文献5篇[1],[3]–[6]，英文文献6篇[7]–[12]，共11例，归纳妊娠合并AT缺乏症的临床情况，见表4。

**表4:** 文献报道的11例妊娠合并抗凝血酶缺乏患者临床特征、处理及转归

文献	患者年龄	孕产史	基因检测	血栓史	血栓后抗凝	孕期抗凝	血浆疗法	产后抗凝	AT活性（％）	妊娠结局
赵璠等[1]	31	G2P1（足月剖宫产24 h内新生儿死亡）		剖宫产术后1周出现左下肢DVT、PE	华法林	LMWH		术后6 h：利伐沙班15 mg/q12h；3周后改为20 mg/qd		孕33周5天剖宫产娩一活婴
卢秋敏等[3]	29	G3P0，稽留流产2次	SERPINC1基因外显子7杂合错义突变	稽留流产后2个月PE	LWMH+华法林；后改为利伐沙班	孕6周：LMWH 6 000 U/q12h+阿司匹林100 mg/qd；孕16周：停用阿司匹林；孕36周：加用华法林3 mg/qd	22周3天：400 ml；3 d后再次输注400 ml	利伐沙班10 mg/qd，产后8个月停药	25～68	孕39周经阴道分娩一男活婴
黄永亮等[4]	29	G1P0	SERPINC1基因杂合变异			LMWH+华法林			20～28	孕17周2天稽留流产
胡丽丽等[5]	27	G2P1		孕10周双下肢DVT、下腔静脉血栓、PE		孕5周：LMWH 6 000 U/q12h；孕12周：加用阿司匹林75 mg/qd；孕21周：阿司匹林100 mg/qd		产后24 h：LMWH4 000 U/q12h		孕38周剖宫产娩一男活婴
于雪飞等[6]	30	G2P0				孕23周：阿司匹林75 mg/qd		产后24 h：LMWH 4 000 U/qd		孕25周4天经阴道分娩一女死婴
Tsikouras等[7]	19	G3P0，早期流产2次				LMWH		ATC+LMWH	33～57	孕39周剖宫产娩一男活婴
Kovac等[8]	23	G1P0	FⅤ Leiden、FⅡ G20210A和MTHFR C677T基因突变			LMWH+ATC		LMWH+口服抗凝（具体不详）	16～33	孕37周经阴道顺娩一男活婴
Pascual等[9]	32	G1P0	SERPINC1基因单碱基对突变	下肢DVT	VKA	孕5周：停用VKA换用LMWH+ATC；孕14周：换用VKA；孕36周：停用VKA，换用LMWH+ATC		产后6 h：LMWH+AT浓缩物；产后3 d：加用VKA；产后10 d：停用LMWH+ATC	40～50	孕37周剖宫产娩一男活婴
Zhang等[10]	32	G3P0	SERPINC1基因杂合突变	早期妊娠脑静脉-静脉窦血栓形成；早期流产后左下肢DVT	华法林	LMWH 4 100U/q12h；孕14周：换用华法林；孕37周：换用LMWH	FFP1单位/周，孕14周停用，孕37周重新启用FFP	LMWH+FFP至产后6周	30～40	孕37周4天剖宫产娩一女活婴
Yamashita等[11]	37	G2P1	SERPINC1基因杂合突变		LMWH	LMWH 10 000U/q12h		LMWH 10 000 U/q12h+华法林2 mg/qd	45	孕38周经阴道分娩一女活婴
Ilonczai等[12]	21	G1P0	FⅤ Leiden基因杂合突变	左下肢DVT	华法林	孕6周：LMWH 30 mg/qd；孕8周：LMWH 40 mg/bid + ATC、华法林		华法林	>26	孕8周自然流产

**注** G：怀孕；P：生产；LMWH:低分子肝素；DVT：下肢深静脉血栓形成；PE：肺栓塞；VKA：维生素K拮抗剂；FFP：新鲜冰冻血浆；ATC：抗凝血酶浓缩物

二、AT缺乏症对妊娠的影响

AT缺乏症为罕见病，发病率为1/20 000～1/2 000[13]–[14]。白种人的发病率高于亚洲和非洲人[1]。AT是体内重要的抗凝物质，主要在肝细胞合成，是丝氨酸蛋白酶抑制物超家族中的一员[15]。该患者存在多次不良孕产史及下肢深静脉血栓形成史，均与AT缺乏症的临床表现相符，如：下肢静脉血栓、颅内静脉血栓、腹腔内静脉血栓，可因体内抗凝失衡导致子宫螺旋动脉或绒毛内微血栓形成，从而导致胎盘灌注不良甚至梗塞，进而导致流产、死胎等不良妊娠结局等[16]–[18]。

三、妊娠对AT缺乏的影响

AT活性正常范围为80％～130％，妊娠早中期无明显变化，妊娠晚期降至基线水平的80％，产时降至基线水平的70％，产后12 h达最低点，产后72 h回升至基线水平[19]。根据静脉血栓发病机制“Virchow”三角学说：静脉血流淤滞、内皮细胞损伤、血液高凝状态，在妊娠期以上3个因素均涉及，如子宫增大导致下肢静脉回流减缓、妊娠期生理高凝、分娩/手术对血管内皮损伤等多种血栓高危因素。

四、AT缺乏症的原因

AT缺乏症为临床罕见病，白种人AT缺乏症的比例为1/5 000～1/500，我国人群遗传性AT缺乏症相关指南尚不完善[20]。根据发病原因分为遗传性AT缺乏症和获得性AT缺乏症。

1. 遗传性AT缺乏症的原因：遗传性AT缺乏症是一种罕见的由于凝血因子遗传性缺陷所导致的常染色体显性疾病，由SERPINC1基因突变引起，是引起血栓形成的重要因素之一[21]。AT缺陷症是1965年由Egeberg[22]首次报道，它的编码基因定位于1q23-25，基因全长13.5 kb，含7个外显子和6个内含子[23]。分为2型：I型（合成减少）主要表现为AT抗原和AT活性的同步减少，为特征的定量障碍；Ⅱ型（功能缺陷）由于AT结构异常所致的功能缺乏，多数AT抗原正常。我院应用法国STAGO全自动凝血分析仪采用发色底物法检测AT活性，本例患者多次检测AT活性为34％～50％，当反复检测确认AT的活性低于正常下限时，可进一步考虑抗原水平检测确定分型。此外，AT缺乏的严重程度和SERPINC1基因突变型相关，且基因检测可帮助鉴别遗传性缺乏还是获得性，故推荐AT活性持续<70％进行基因检测[24]。本例患者进行了基因检测，第1次检测涵盖了58个血栓遗传基因，未发现抗凝血酶全长基因异常，排除了遗传性AT缺乏。

2. 获得性AT缺乏症的原因：①合成减少，如营养不良、肝功能障碍等；②排泄增加，如肾病综合征、糖尿病；③消耗增加，如急性或近期静脉血栓栓塞、弥散性血管内凝血、败血症、手术、创伤、大出血、子痫前期、怀孕、产后、溶血性尿毒症综合征、全身炎症反应综合征；④药物引起的消耗，如肝素、口服避孕药、门冬酰胺酶；⑤体外循环，如血液透析、血浆置换、体外循环、体外膜肺氧合[25]。本例无营养不良，肝功能正常，无合成减少证据；孕期血糖、血压正常，无肾病综合征、糖尿病、子痫前期，无排泄增加的证据；孕30周4天下肢血管超声提示血流通畅，无血栓形成，无消耗增加；未口服避孕药物，无体外循环，无药物或体外循环影响，获得性降低的原因考虑可能为妊娠或肝素，但患者备孕未用肝素时查AT活性为41％，2020年孕26周未抗凝时查AT活性为21％，本次妊娠产后停肝素1周查AT活性为44％，故AT活性降低无法用肝素的影响或妊娠解释。检测Ⅷ因子活性（48.9％）明显降低，考虑可能与AT为适应Ⅷ因子下降、凝血与抗凝在低水平维持平衡有关。

3. AT的抗凝机制：AT能使凝血酶（Ⅱa因子）以及凝血因子Ⅸa、Ⅹa、Ⅺa和Ⅻa失活，肝素作用下，AT抑制凝血酶的活性增强1 000倍以上[26]。AT包括两个主要的功能区：位于N端的肝素结合区和位于C端的反应位点[27]。SERPINC1基因作为AT的编码基因，具有高度保守的二级结构和反应中心环，可通过与蛋白酶活性位点的相互作用来抑制蛋白酶活性。

4. AT缺乏症的抗凝选择：关于妊娠期抗凝药物及剂量选择，目前缺少大样本临床数据研究，缺乏统一的抗凝方案，需要产科和血液科共同管理，以降低血栓发生风险，及时调整抗凝用药剂量，提高AT水平，改善妊娠结局。常用抗凝药物：①华法林：维生素K拮抗剂，该药物可通过胎盘导致胎儿出血、致畸等。②低分子肝素：其主要通过增强AT与凝血酶的亲和力阻止内源性凝血途径发挥作用，该药物不能通过胎盘。故本例患者初期给予低分子肝素抗凝治疗，效果欠佳。③利伐沙班：Ⅹa因子抑制剂，动物研究表明有生殖毒性，禁用于孕妇。④达比加群酯：直接抑制凝血酶，Ⅱa因子抑制剂，动物研究表明有生殖毒性，排除特殊情况使用，妊娠女性不应接受本品治疗。根据2018年美国妇产科医师协会（American college of obstetricians and gynecologists，ACOG）指南[28]，妊娠期抗凝药物首选普通肝素和低分子肝素。用AT活性水平判断肝素是否有效，AT活性<70％时，肝素作用降低65％；<50％时，肝素作用为原来的1/5，提示需要补充；<30％时，提示肝素抗凝无效。本例患者孕期全程使用依诺肝素抗凝，鉴于AT活性为34％～50％，为提高低分子肝素抗凝效果亟需提高AT活性，可选择血浆来源的AT、人重组AT浓缩物和FFP[29]。我国尚未批准前2种AT制剂，本例患者只能通过输注FFP补充AT，3次血浆输注后AT活性上升分别为：12％、6％、7％，提示短期内多次输注反而可能通过激活机体的免疫反应从而加速对外源性AT的破坏，因此，如何慎重选择血浆输注时机、充分发挥FFP的最大效应，还有待进一步研究。

综上所述，目前AT缺乏症的研究缺乏大样本临床数据，妊娠期AT缺乏症的管理主要基于病例报道和专家意见。早期诊断、规范治疗可显著改善妊娠结局。
